# Tissue-Specific Accumulation of Sulfur Compounds and Saponins in Different Parts of Garlic Cloves from Purple and White Ecotypes

**DOI:** 10.3390/molecules22081359

**Published:** 2017-08-20

**Authors:** Gianfranco Diretto, Angela Rubio-Moraga, Javier Argandoña, Purificación Castillo, Lourdes Gómez-Gómez, Oussama Ahrazem

**Affiliations:** 1 Italian National Agency for New Technologies, Energy and Sustainable Development, Casaccia Research Centre, 00123 Rome, Italy; gianfranco.diretto@enea.it; 2 Instituto Botánico, Departamento de Ciencia y Tecnología Agroforestal y Genética, Facultad de Farmacia, Universidad de Castilla-La Mancha, Campus Universitario, s/n, 02071 Albacete, Spain; angela.rubio@uclm.es (A.R.-M.); Javier.argandona@uclm.es (J.A.); marialourdes.gomez@uclm.es (L.G.-G.); 3 Coopaman S.C.L., Departamento I+D, Carretera Peñas De San Pedro, km 1.6, 02006 Albacete, Spain; culviman@coopaman.com; 4 Facultad de Ciencias Ambientales y Bioquímica, Universidad de Castilla-La Mancha, Campus Tecnológico de la Fábrica de Armas, Avda, Carlos III, s/n, E-45071 Toledo, Spain

**Keywords:** purple and white garlic, sulfur compounds, saponins, LC-HRMS, correlation networks

## Abstract

This study set out to determine the distribution of sulfur compounds and saponin metabolites in different parts of garlic cloves. Three fractions from purple and white garlic ecotypes were obtained: the tunic (SS), internal (IS) and external (ES) parts of the clove. Liquid Chromatography coupled to High Resolution Mass spectrometry (LC-HRMS), together with bioinformatics including Principal Component Analysis (PCA), Hierarchical Clustering (HCL) and correlation network analyses were carried out. Results showed that the distribution of these metabolites in the different parts of garlic bulbs was different for the purple and the white ecotypes, with the main difference being a slightly higher number of sulfur compounds in purple garlic. The SS fraction in purple garlic had a higher content of sulfur metabolites, while the ES in white garlic was more enriched by these compounds. The correlation network indicated that diallyl disulfide was the most relevant metabolite with regards to sulfur compound metabolism in garlic. The total number of saponins was almost 40-fold higher in purple garlic than in the white variety, with ES having the highest content. Interestingly, five saponins including desgalactotigonin-rhamnose, proto-desgalactotigonin, proto-desgalactotigonin-rhamnose, voghieroside D1, sativoside B1-rhamnose and sativoside R1 were exclusive to the purple variety. Data obtained from saponin analyses revealed a very different network between white and purple garlic, thus suggesting a very robust and tight coregulation of saponin metabolism in garlic. Findings in this study point to the possibility of using tunics from purple garlic in the food and medical industries, since it contains many functional compounds which can be exploited as ingredients.

## 1. Introduction

Plants produce hundreds of thousands of different natural products known as secondary metabolites. These small organic molecules allow plants to withstand different types of environmental conditions and stresses [1]. The functions of secondary metabolites in plants have not been adequately elucidated, although it is known that plants invest many resources in synthesizing, storing and accumulating these compounds, which cover a vast range of ecophysiological functions. Often, these metabolites are produced through complex and highly regulated biosynthetic pathways [2]. The species *Allium sativum* L., commonly known as garlic, belongs to the genus *Allium* and represents one of the most numerous and important groups of the Alliaceae subfamily. It comprises, around 450 species, which are widely distributed in the northern hemisphere. All the species differ in their ripening characteristics, color, and flavor, which are mainly determined by their biochemical properties, consequently affecting their phytochemical content and nutraceutical effects.

Commercial garlic is grouped into two species: *Allium sativum* var. *sativum* and *A. sativum* var. *ophioscorodon*, although other species are also cultivated in minority for culinary uses, e.g., *Allium porrum* L., *Allium fistulosum* L., *Allium ascalonicum* Hort., *Allium ursinum* L., *Allium ampeloprasum* L. var. *Ampeloprasum*, *Allium schoenoprasum* L., and *Allium tuberosum* L. The taste, average storage life and the ability to grow in diverse environments are determining traits for novel garlic varieties to be commercialized.

Consumption of garlic is traditional in many countries as a culinary additive. References to culinary and medicinal uses date back to Sumerian times (2600–2100 BCE) [3]. It reportedly has antioxidant, anti-inflammatory, and antimicrobial properties. These characteristics allow raw and processed garlic to be used successfully in treating and preventing a wide range of diseases such as cardiovascular problems and cancers [4,5].

The chemistry of *Allium* species has been dominated by sulfur-containing compounds known as allicins, which give them their distinctive flavor and aroma. However, different compounds, including those lacking sulfur, act synergistically to provide different health benefits such as fructans [6], flavonoids [7] and saponins [8]. Numerous preclinical and in vitro studies have shown that allicin-free garlic products have clear and significant biological effects in preventing tumors, in stimulating immune responses, protecting the liver, etc. The *S*-allyl cystein (SAC) is the precursor of all the sulfur compounds (whose biosynthesis is shown in Figure S1A). The first intermediate produced is alliin, which is subsequently converted to allicin through the action of the alliinase enzyme. It is from allicin that all the other sulfur compounds (ajoene, diallyl sulfide (DAS), disulfide (DADS), etc.) are then generated. On the contrary, the synthesis of saponins occurs through the squalene precursor, until the generation of β-chlorogenin, diosgenin and gitogenin, which represent the building blocks for all the garlic saponins (desgalactotigonin-, eruboside B-, sativoside- and voghieroside-types) (Figure S1B).

The main purpose of this study was to analyze the distributions of sulfur compounds and saponins in different sections of bulbs from purple garlic (var. Morado de Cuenca) and from white garlic (var. Porcelain) in order to highlight ecotype- and tissue-specificity in relation to the accumulation of high-value compounds. Furthermore, the possibility of utilizing nonedible parts for industrial purposes, by means of the determination of their bioactive metabolic pools, has been investigated.

## 2. Results and Discussion

In order to determine the distribution of sulfur and saponin metabolites throughout the different parts of garlic bulbs, two ecotypes were chosen: purple (var. Morado de Cuenca) and white garlic (var. Porcelain) since they represent important commercial varieties in Spain.

**Figure 1:**
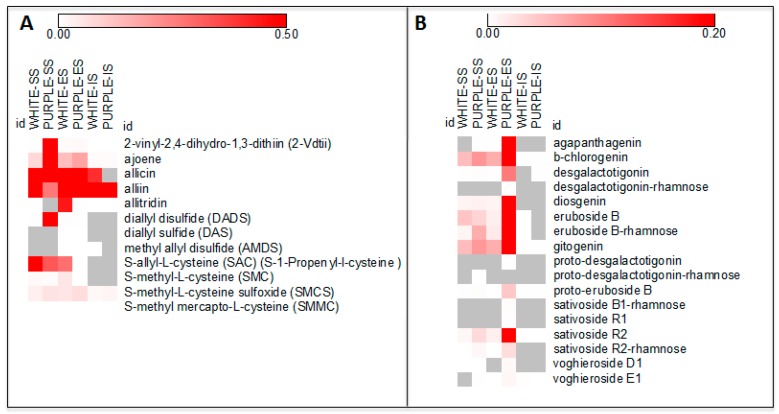
Heatmap visualization of sulfur compounds (**A**) and saponins (**B**) detected in three tissues (SS, ES and IS) of the white and purple ecotypes. Data represent, for each metabolite, the fold over the internal standard (IS) intensity.

To investigate the sulfur-containing compounds and saponins present in the two mentioned ecotypes, Liquid Chromatography coupled to High Resolution Mass (LC-HRMS) analyses were performed. A total of 12 sulfurs and 17 saponin compounds were determined and are listed in Table 1, Table 2, Table S1 and Figure 1.

The 12 sulfur metabolites found were 2-vinyl-2,4-dihydro-1,3-dithiin (2-Vdtii), ajoene, allicin, alliin, allitridin, diallyl disulfide (DADS), diallyl sulfide (DAS), methyl allyl disulfide (AMDS), *S*-allyl-l-cysteine (SAC) (*S*-1-Propenyl-l-cysteine), *S*-methyl-l-cysteine (SMC) *S*-methyl-l-cysteine sulfoxide (SMCS) and *S*-methyl mercapto-l-cysteine (SMMC), were detected in both varieties. In our experimental conditions, allicin and alliin constituted the sulfur compounds showing the highest signal intensities (Figure 1A).

The total of the sulfur compounds obtained was slightly higher in purple than in white garlic (Table 1). Both ecotypes shared the same metabolites, but with different proportions.

The metabolites in diallyl disulfide (DADS), 2-vinyl-2,4-dihydro-1,3-dithiin (2-Vdtii), ajoene, allicin, *S*-methyl-l-cysteine sulfoxide (SMCS) and *S*-methyl mercapto-l-cysteine (SMMC) were higher in purple garlic than in white (234.90-, 158.70-, 13.86-, 2.25-, 1.85- and 1.44-fold, respectively), while allidrin (111.71), alliin (2.09), diallyl sulfide (DAS) (1.39), *S*-allyl-l-cysteine (SAC) (*S*-1-Propenyl-l-cysteine) (2.44) and *S*-methyl-l-cysteine (SMC) (8.63) were shown to be in higher proportions in white than in purple garlic. The distribution of these metabolites throughout the different parts of garlic cloves was different between the purple and white ecotypes. A higher amount of sulfur compounds was found in the SS and ES of purple and white garlic, respectively, while the lower values were obtained in the IS fraction in both ecotypes (Table 2 and Table S1).

Ajoene and allicin followed the same pattern, with higher contents in purple SS and ES tissues compared to the white variety, while displaying the opposite behavior in IS tissues. Alliin was found 22.53-fold higher in SS from white garlic than in the purple cultivar, with similar trends detected in the ES and IS parts. Another well-known sulfur compound, allitridin, was detected in white SS but not in purple SS tissues. Furthermore, it was found to be more highly accumulated in the ES part of the white than in the purple ecotype (115.04-fold), while it was detected at a higher level in IS purple, compared to the white one (1.69-fold) (Table 2).

Regarding the allyl sulfide group, diallyl disulfide (DADS) was differentially accumulated in all parts of the varieties, but with a distinct tissue-specificity. In the SS part, it accumulated 758.09-fold in purple over the white variety, while an opposite trend was observed in ES and IS tissues (2.43- and 4.77-fold, respectively). Diallyl sulfide (DAS) was only found in ES (1.39-fold in white compared to the purple ecotype), while methyl allyl disulfide (AMDS) was detected in ES parts, although at no different level between the two genotypes, and in the IS tissue of the white ecotype. The *S*-methyl-l-cysteine sulfoxide (SMCS) and *S*-methyl mercapto-l-cysteine (SMMC) were measured in SS tissues at higher contents in purple vs white variety (3.93 and 1.71-fold), and with opposite behavior in IS parts (2.80- and 1.36-fold in the white ecotype over the purple one). Finally, 2-vinyl-2,4-dihydro-1,3-dithiin (2-Vdtii) was 638.66-, 1.44- and 1.68-fold higher in the SS, ES and IS of the purple cloves than in the white cloves, respectively (Table 2). Interestingly, two well-known sulfur compounds associated to garlic aging [9], *S*-allyl-l-cysteine (SAC) (*S*-1-Propenyl-l-cysteine) and *S*-methyl-l-cysteine (SMC), were not detected in IS tissues in either ecotype, while higher amounts were found in the SS and, especially, in the ES tissues.

The main compound found in whole garlic cloves is alliin, which accounts for approximately 80% of cysteine sulfoxides. The allicin (diallyl thiosulfinate) responsible for garlic’s pungent smell does not exist in raw garlic until the cloves are crushed, chopped, or chewed, thus releasing the alliinase enzyme [10]. This enzyme in intact tissues is compartmentalized within plant vacuoles and catalyzes, in the presence of the cofactor pyridoxal 5′-phosphate, the formation of pyruvate, ammonia and sulfenic acids, which spontaneously react with each other to form unstable compounds called thiosulfinates, and known as allicins. The formation of allicins is a very fast reaction completed within 10 to 60 s following crushing. Allicin is metabolized in vitro to form a variety of fat-soluble sulfur compounds, including diallyl trisulfide (DATS), diallyl disulfide (DADS), and diallyl sulfide (DAS) or, in the presence of oil or organic solvents, ajoene and vinyldithiins. In vivo, allicin can react with glutathione and l-cysteine to produce *S*-allylmercaptoglutathione (SAMG) and *S*-allylmercaptocysteine (SAMC), respectively.

Sulfur compounds are also responsible for the medicinal properties of garlic, such as immunomodulatory, anti-inflammatory, antidiabetic, antimicrobial, cardioprotective, antioxidant, and anticancer activities [11,12,13].

Among the 12 sulfur compounds, the most interesting metabolites are alliin, allicin and diallyl disulfide (DADS). Alliin has been described as an antioxidant and can inhibit the damaging effects caused by free-radical agents, including reactive oxygen, nitrogen, and chlorine species [14], while allicin has shown antimicrobial properties against both Gram-positive, Gram-negative and acid-fast bacteria [15,16]. Diallyl disulfide (DADS)has been reported as a potent anticancerogenic agent, reducing the levels of chemically induced tumors in rodent models [17]. Different studies have shown that it can act as an inductor of antioxidant responsive elements, or can inhibit different enzymes such as the Cytochrome P450 monoxygenases, especially the CYP450 2E1-dependent bioactivation of procarcinogens [18]. Additionally, diallyl disulfide(DADS) can also promote the initiation of apoptosis [19].

Results obtained in the present study on sulfur compounds showed that, even though in both cultivars the same metabolites have been detected, it seems that the purple variety is highly enriched in metabolites involved in preventing damages against human health. The distribution of these compounds is different between the two varieties, with the SS fraction from the purple garlic being the highest one in terms of sulfur metabolite contents in comparison to the other fractions, whereas the ES of white garlic constitutes the more enriched one in these compounds.

**Figure 2:**
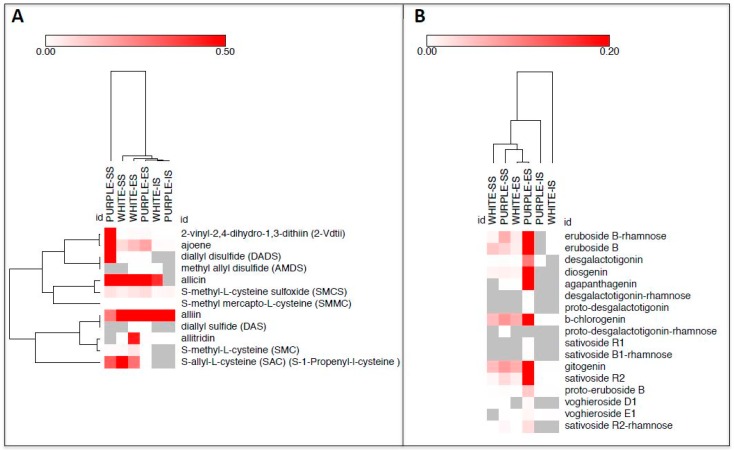
Hierarchical clustering (HCL) visualization, applied to rows and columns, of sulfur compounds (**A**) and saponins (**B**) detected in three tissues (SS, ES and IS) of the white and purple ecotypes of garlic. Data represent, for each metabolite, the fold over the internal standard (IS) intensity.

**Figure 3:**
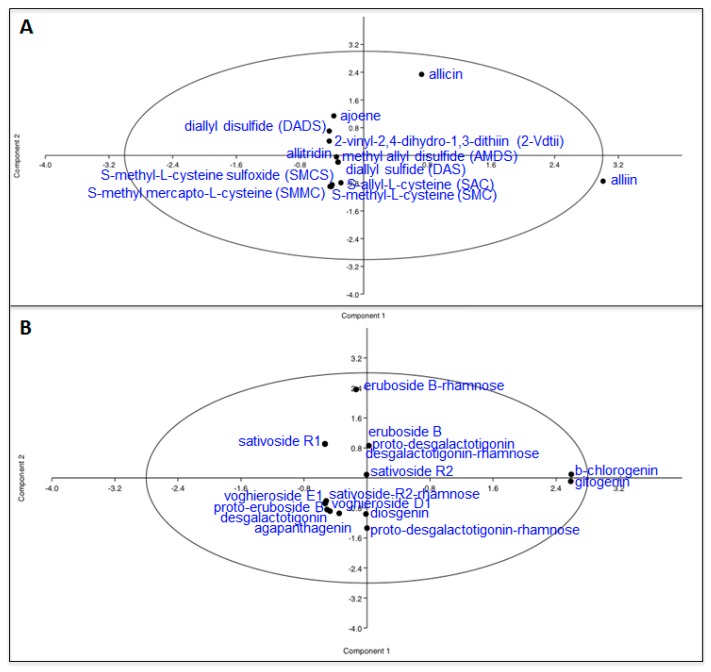
Principal component analysis (PCA), according to the metabolites, of sulfur compounds (**A**) and saponins; (**B**) detected in three tissues (SS, ES and IS) of the white and purple ecotypes of garlic. Data represent, for each metabolite, the fold over the internal standard (IS) intensity.

**Figure 4:**
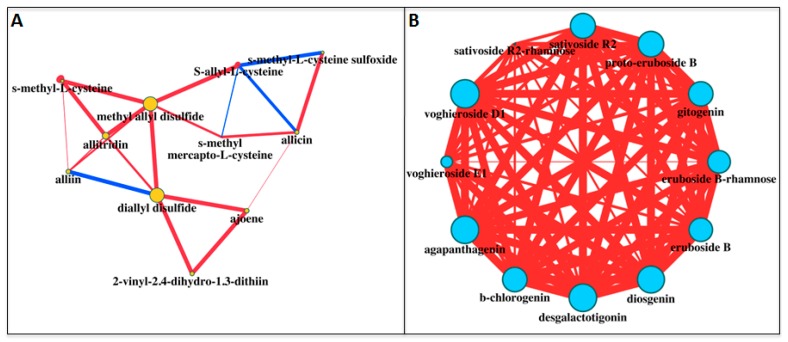
Correlation networks of sulfur compounds (**A**) and saponins (**B**) detected in three tissues (SS, ES and IS) of the white and purple ecotypes of garlic. Pearson correlation (ρ) coefficients were calculated for each metabolite pair. Node colors identify sulfur compounds (light orange, left) and saponins (light blue), while node size was proportional to node strength (ns). Red and blue edges refer to, respectively, positive and negative correlations. Edge thickness was according to the corresponding |ρ|. Only ρ >|0.50| were shown.

We used a series of bioinformatic analyses to better elucidate the accumulation of sulfur compounds in the three tissues of the white and purple ecotypes. First of all, we applied the Pearson squared distance algorithm in a hierarchical clustering (HCL) analysis (Figure 2A), highlighting the relevance of SS tissue of the purple ecotype, which did not cluster with any of the other samples. On the contrary, the ES and IS parts of the two ecotypes clustered together. Clustering applied on the rows identified two distinct groups made up of 2-vinyl-2,4-dihydro-1,3-dithiin (2-Vdtii), ajoene, allicin, *S*-methyl-l-cysteine sulfoxide (SMCS) and *S*-methyl mercapto-l-cysteine (SMMC) (top side of the HCL) and alliin, diallyl sulfide (DAS), allitridin, diallyl disulfide (DADS), methyl allyl disulfide (AMDS). Principal component analysis (PCA) was also performed to identify the compounds mainly discriminating the tissues/ecotypes (Figure 3A). Allicin and alliin were found to be the main metabolites along with, respectively, component 1 and 2 in the PCA. When applied to the tissues/ecotypes (Figure S2A), the analysis did not allow a clear separation, although, in agreement with the previous findings, SS tissue from the purple garlic localized more distantly from the other experimental points. Finally, a correlation network was drawn by computing, for each sulfur compound pair, the corresponding Pearson correlation coefficient (ρ) (Figure 4A). All the correlations observed were positive, except for the one between diallyl sulfide (DAS) and alliin, which acts as its precursor; the ones within *S*-allyl-l-cysteine (SAC) towards allicin and *S*-methyl mercapto-l-cysteine, and the one between *S*-methyl-l-cysteine sulfoxide (SMCS) and *S*-allyl-l-cysteine (SAC). Nodes in the network displayed different sizes, defined as node strength (ns, representing the mean of all the ρ passing for each node). In this context, interestingly, diallyl disulfide (DADS) was the node showing the highest node strength (ns), which could indicate a more relevant role in the frame of the sulfur compound metabolism in garlic.

Besides the values and the biological activities of the sulfur components, there are other compounds present in garlic with potential bioactive compounds such as saponins. An increasing number of studies has described saponins as powerful antifungal, cytotoxic, anti-inflammatory, antithrombotic and hypocholesterolemic molecules [11,20]. Furthermore, a strong ability to remove excess cholesterol from blood has been assigned to these molecules [21]. Saponins are generally divided into two groups, triterpenoid saponins and steroid saponins, based on the molecular structure of the aglycone. The steroid saponins are further subdivided into three groups: spirostanols, furostanols and cholestanes [22]. In garlic, the spirostanol saponins are obtained by enzymatic activity of β-glucosidases over furostanols. The processing of garlic not only leads to variations in the amounts and types of organosulfur compounds but also to variations in steroid saponins.

In this study, 17 steroid saponins belonging to the spirostanol and furostanol groups have been identified and reported in Table 1, Table 2 and Table S2. Twelve of them were detected in both varieties: agapanthagenin, β-chlorogenin, desgalactotigonin, diosgenin, eruboside B, eruboside-B-rhamnose, gitogenin, proto-eruboside B, sativoside B1-rhamnose, sativoside R2, sativoside R2-rhamnose, and voghieroside E1. Interestingly, five saponins (desgalactotigonin-rhamnose, proto-desgalactotigonin, proto-desgalactotigonin-rhamnose, voghieroside D1, sativoside B1-rhamnose and sativoside R1) were exclusive to the purple variety and were present in the ES tissue, except for the proto-desgalactotigonin-rhamnose, which was also found in the SS fraction. In our experimental conditions, β-chlorogenin, eruboside B, eruboside B-rhamnose and gitogenin were the saponins with the highest signal intensities (Figure 1B).

The total number of saponins (Table 1) was almost 40-fold higher in purple garlic than in the white variety. The distribution of these metabolites showed a difference between the two ecotypes. In white garlic, in fact, the same level of saponins was detected in SS and ES, while traces were found in the IS fraction. On the contrary, in purple garlic, the highest content was obtained in the ES, followed by the SS, while only traces were detected in the IS fraction (Table S2).

The most common saponins identified in *Allium* plants are diosgenin, gitogenin and β-cholorogenin. However, the content of these saponins in plants is usually low, except for *A. nutans*, where very high concentrations, up to 4% of the dry material, have been reported [22]. Our results showed that white garlic accumulated low levels of saponins, findings already reported in other allium species such as *A. ursinum* [23] and *A. nigrum* [24], whereas purple garlic displayed a content similar to those described for *A. nutans* [25] (Table S2). Nevertheless, when comparing the two ecotypes, the purple variety revealed a tremendous increase compared to the white one, particularly in ES tissues (β-chlorogenin: 76.99; diosgenin, 95.30) (Table 2). Gitogenin, sativoside R2 and sativoside R2-rhamnose (which was not detected in IS tissues) showed the same pattern of β-chlorogenin, being over-accumulated in purple SS and ES parts over the white variety. Agapanthagenin, a saponin with specific antispasmodial activity [26] was also present at higher contents in the purple ecotype, both in SS (where it was not found in the white ecotype) and in ES (320.25-fold).

One of the most interesting steroid saponins is eruboside B, with a formation similar to allicin, since it is also obtained when raw garlic is crushed, and appears after the enzymatic transformation from the glycoside proto-eruboside. In our experimental conditions, eruboside B resulted in, slightly over-accumulated in the SS parts in white over the purple ecotype (1.30). On the contrary, in ES tissues, a 64.09-fold accumulation was found in purple vs white garlic. In addition, eruboside B-rhamnose, a glucosidic derivative of eruboside B, showed higher accumulation in SS (8.33-fold) and in ES (37.38-fold) parts. Notably, a different trend was observed for the proto-eruboside B, displaying a higher level in the purple genotype both in SS (5.87) and in ES (293.71) tissues (Table 2). In addition, proto-eruboside B was also identified in the IS parts, with higher levels found in white vs purple ecotypes (2.05). Eruboside B has been described as an inhibitor of the growth of *Candida albicans*, and its activity is comparable to that of allicin and ajoene, while the precursor proto-eruboside B does not possess any antifungal activity [27]. Furthermore, eruboside B isolated from *Allium chinense* has exhibited, in vitro, a strong antitumoral activity against the B16 melanoma and 4T1 breast carcinoma cell lines and, in vivo, towards the growth of melanoma [28]. In the framework of the garlic steroidal saponin dataset under investigation, we were also able to detect desgalactotigonin and its precursor, proto-desgalactotigonin, alone or conjugated with rhamnose. Recently, desgalactotigonin has attracted much interest for its spermicidal and contraceptive activity [29]. In our experimental conditions, desgalactotigonin was detected at higher levels in the purple SS (5.09-fold) and ES (98.53-fold) parts, while desgalactotigonin-rhamnose was only found in the purple ES tissue. Additionally, proto-desgalactotigonin and proto-desgalactotigonin-rhamnose accumulated in, respectively, purple ES and SS, indicating a very active desgalactotigonin and proto-desgalactotigonin metabolism in garlic. Two saponins with a strong antifungal bioactivity, vogheroside D1 and E1 [30], were measured in garlic samples. While the former was detected in purple but not in white ES tissue, resulting in an over-accumulation in the purple ecotype (4.56-fold), the latter was found at higher levels in purple ES and white IS (67.90- and 5.11-fold, respectively). Finally, sativoside B1-rhamnose and R1 were only found in the ES tissues of the purple ecotype.

Similarly to that done with sulfur compounds, we applied the same bioinformatic approaches to the saponin dataset. HCL revealed the particularity of IS tissue of the white ecotype as “outlier” of the dataset, since it was placed at the far right of the HCL (Figure 2B). Simultaneously, metabolites on the rows clustered, in most of cases, according to their subgroup: eruboside B and eruboside B-rhamnose at the top of the HCL; sativoside R1 and sativoside B1-rhamnose, vogheroside D1 and vogheroside E1 in, respectively, the median and the lower area of the HCL. PCA, according to the metabolites, indicated β-chlorogenin and gitogenin, and eruboside B-rhamnose, as mainly responsible for the dataset variance throughout the components 1 and 2 of the PCA, respectively (Figure 3B). On the contrary, when applied to the samples, only the ES tissue of the purple ecotype slightly separated along the component 1 (Figure S2B). Correlation analyses of saponins in white and purple garlic evidenced a very different network compared to the sulfur compounds. Here, in fact, most of the correlations were highly significant (>0.97) and of a positive sign, indicating a very robust and tight coregulation of saponin metabolism in garlic (Figure 4B). In agreement with this hypothesis, most of the nodes, with the exception of sativoside R2-rhamnose and voghieroside E1, were characterized by similar ns values.

The role of saponins in plant defense has been broadly suggested, and their in vitro antifungal activity versus several plant pathogens has been previously demonstrated [31]. Hence, it is not surprising that these metabolites were accumulated in the SS and ES parts, instead of in the IS fraction, since they could act as a first barrier to fight pathogen attacks. The fact that the different garlic cultivars showed significant differences in susceptibility to the infections by fungi, with white garlic varieties more sensitive than other ecotypes [32], reinforces the hypothesis that saponins, together with other metabolites, can play an essential role in the defense against pathogens in garlic.

## 3. Materials and Methods

### 3.1. Plant Materials

The plant materials, purple (var. Morado de Cuenca) and white (var. Porcelain) garlic, used in this study were provided by Coopaman S.C.L at the ripening stage (Figure S3). The crops were cultivated on irrigated neighboring land, management was carried out to provide optimum plant growth and yield including fertilization, plant protection treatment and irrigation according to standards of the region. Three different fractions have been separated; the tunic (SS) was separated from the cloves by hand, the cloves were dissected into two parts: the external (ES) part, corresponding to the peel fraction; and the internal (IS) part, corresponding to the tissue from which the leaves will emerge after dormancy. After sampling, the materials were lyophilized and kept at −80 °C until used.

### 3.2. HPLC-MS

Extraction of semipolar and metabolites was performed as previously described [33,34,35]. Briefly, 10 mg of homogenized garlic powder were, with 0.75 mL cold 75% (*v*/*v*) methanol, 0.1% (*v*/*v*) formic acid, spiked with 10 μg/mL of formononetin. After shaking for 20 min + 20 min (with a break of 10 min) at 20 Hz using a Mixer Mill 300 (Qiagen, Venlo, Netherlands), samples were centrifuged for 15 min at 20,000 *g* at 10 °C; 0.6 mL of supernatant were then removed and transferred to High Performance Liquid Chromatography (HPLC) tubes (Phenomenex, Torrance, CA, USA). For each experimental point, at least two technical replicates (independent extractions) from three biological replicates (independent pools), with a total of six chromatograms/experimental point were performed. All solvents were HPLC grade and were purchased from Sigma-Aldrich (Steinheim, Germany). Liquid Chromatography coupled to High Resolution Mass Spectrometry (LC-HRMS) was carried out with slight modifications limited to the mass spectrometry part and was performed immediately after extraction to avoid enzymatic degradation of the sample. More detailed, electrospray ionization (ESI) was exploited to determine the sulfur compounds and saponins, and was achieved by using the following parameters: capillary temperature set at 290 °C; sheath, aux and sweep gas flow rates at 25, 10 and 0 L/min, respectively. Spray voltage was 3.0 kV, while capillary voltage and tube lens were set at, respectively, 35 and 90 V. Metabolite identification was obtained by comparing chromatographic and MS properties of target compounds with authentic standards, if available; on the basis of the *m*/*z* accurate masses, as reported in the PubChem database for monoisotopic mass identification, or in the Metabolomics Fiehn Lab Mass Spectrometry Adduct Calculator for adduct ion detection; and, on the basis of mass fragmentation, as reported in Metlin (http://metlin.scripps.edu/landing_page.php?pgcontent=mainPage), or by comparing theoretical and experimental mass fragmentation with the software MassFrontier 7.0 (Thermo Fisher Scientific, Waltham, MA, USA). Metabolite quantification for each compound was performed relative to the internal standard values.

### 3.3. Bioinformatics

Analysis of Variance (ANOVA), Tukey’s *t*-test and Principal Component Analysis (PCA) were performed by using PAST [36]. Hierarchical clustering and correlation networks were carried out as previously reported [37,38]. Briefly, hierarchical clustering was performed on data tables organized as follows: each row representing a metabolite, columns for the three tissues of the white and purple ecotypes of garlic, and data points showing the associated log2-fold changes. Correlation networks were assembled manually from the corresponding matrices. Each data pair in the matrix was converted to a single line (edge) connecting two nodes. In the network diagrams, edge thickness is proportional to the absolute value of the Pearson correlation coefficient (|ρ|), while node sizes are proportional to their node strengths. Positive (ρ > 0) and negative (ρ < 0) correlations are shown in red and blue, respectively.

## 4. Conclusions

Our results indicated different distribution patterns of the sulfur and saponin metabolites between the studied ecotypes, with some of them displaying distinct cultivar-tissue-specificity. Overall, it appears that the purple cultivar has more pharmacological benefits than the white ecotype, since it contains a higher saponin content localized mainly in the tunic and the external part of the cloves. Thus, to take advantage of all the benefits of the purple cultivar, garlic should be consumed without discarding the clove tunics since they contain the most important molecules that are responsible for the properties of garlic. Alternatively, these findings provide a new opportunity for utilizing garlic crop parts which are not edible and constitute undesirable residues. These can be exploited as alternative sources of high-value metabolites in novel pharmacological formulations.

## Figures and Tables

**Table 1:** LC-HRMS analyses of sulfur compounds and saponins in white and purple garlic ecotypes. Data are expressed as Average ± Standard Deviation (AVG±ST.DEV), and as fold between the two garlic varieties. AVGs, which represent, for each metabolite, the fold over the internal standard (IS), have been obtained by using at least 3 independent biological replicates. Asterisks indicate significance of the fold change in an analysis of variance (ANOVA) plus Tukey’s *t*-test (*: *p* ≤ 0.05; **: *p* ≤ 0.01; ***: *p* ≤ 0.001).

**Sulfur Compounds**	**White**	**Purple**	**White/Purple**	**Purple/White**
2-vinyl-2,4-dihydro-1,3-dithiin (2-Vdtii)	0.0115 ± 0.0016	1.8250 ± 0.0011 ***	0.0063	158.7012
Ajoene	0.2148 ± 0.0089	2.9764 ± 0.0161 ***	0.0722	13.8574
Allicin	3.1560 ± 0.1577	7.1073 ± 0.3014 ***	0.4440	2.2520
Alliin	14.3933 ± 0.3834 ***	6.8875 ± 0.1465	2.0898	0.4785
Allitridin	0.4451 ± 0.0002 ***	0.0040 ± 0.0005	111.7082	0.0090
Diallyl disulfide (DADS)	0.0095 ± 0.0001	2.2228 ± 0.0002 ***	0.0043	234.9036
Diallyl sulfide (DAS)	0.0007 ± 0.0001	0.0005 ± 0.0001	1.3896	0.7196
Methyl allyl disulfide	0.0002 ± 0.0001	0.0001 ± 0.0001	1.2389	0.8071
*S*-Allyl-l-cysteine (SAC) (*S*-1-Propenyl-l-cysteine)	0.8031 ± 0.1176 ***	0.3293 ± 0.1009	2.4388	0.4100
*S*-Methyl-l-cysteine (SMC)	0.0630 ± 0.018 ***	0.0073 ± 0.009	8.6301	0.1159
*S*-Methyl-l-cysteine sulfoxide (SMCS)	0.1006 ± 0.0146	0.1450 ± 0.0096	0.6941	1.4406
*S*-Methyl mercapto-l-cysteine	0.0010 ± 0.0001	0.0019 ± 0.0001 *	0.5412	1.8477
Total	19.1960 ± 0.5887	21.5063 ± 0.4919 *	0.8926	1.1204
**Saponins**	**White**	**Purple**	**White/Purple**	**Purple/White**
Agapanthagenin	0.0009 ± 0.0002	0.2938 ± 0.0212 ***	0.0031	321.3416
β-chlorogenin	0.1201 ± 0.0279	5.1217 ± 0.5347 ***	0.0234	42.6560
Desgalactotigonin	0.0015 ± 0.0003	0.1077 ± 0.0120 ***	0.0144	69.4736
Desgalactotigonin-rhamnose	nd	0.0018 ± 0.0001	0.0000	-
Diosgenin	0.0174 ± 0.0039	0.8497 ± 0.0515 ***	0.0204	48.9731
Eruboside B	0.0604 ± 0.0103	0.9349 ± 0.1731 ***	0.0646	15.4892
Eruboside B-rhamnose	0.0249 ± 0.0036	0.7043 ± 0.1750 ***	0.0353	28.2983
Gitogenin	0.1199 ± 0.0279	5.1086 ± 0.5335 ***	0.0235	42.6249
Proto-desgalactotigonin	0.0001 ± 0.0001	0.0004 ± 0.0001 *	0.1634	6.1215
Proto-desgalactotigonin-rhamnose	nd	0.0003 ± 0.0001	0.0000	-
Proto-eruboside B	0.0016 ± 0.0001	0.0475 ± 0.0115 ***	0.0327	30.5637
Sativoside B1-rhamnose	nd	0.0026 ± 0.0002	0.0000	-
Sativoside R1	nd	0.0014 ± 0.0004	0.0000	-
Sativoside R2	0.0199 ± 0.0022	0.8814 ± 0.2130 ***	0.0226	44.2229
Sativoside R2-rhamnose	0.0004 ± 0.0001	0.0337 ± 0.0079 ***	0.0121	82.5336
Voghieroside D1	0.0001 ± 0.0001	0.0049 ± 0.0008 ***	0.0118	84.6999
Voghieroside E1	0.0017 ± 0.0001	0.0082 ± 0.0022 **	0.2042	4.8961
Total	0.3687 ± 0.0765	14.1029 ± 1.7371 ***	0.0261	38.2539

**Table 2:** Fold level of sulfur compounds and saponins in three tissues (tunic (SS), external part (ES) and internal part (IS)) from white and purple ecotypes.

	**SS**		**ES**		**IS**	
**Sulfur Compounds**	**White/Purple**	**Purple/White**	**White/Purple**	**Purple/White**	**White/Purple**	**Purple/White**
2-vinyl-2,4-dihydro-1,3-dithiin (2-Vdtii)	0.0016	638.6639	0.6930	1.4431	0.5953	1.6798
Ajoene	0.0271	36.8755	0.7260	1.3774	0.8480	1.1793
Allicin	0.1559	6.4160	0.5931	1.6861	-	-
Alliin	22.5323	0.0444	1.2892	0.7757	1.1267	0.8875
Allitridin	-	-	115.0373	0.0087	0.5913	1.6913
Diallyl disulfide (DADS)	0.0013	758.0864	2.4338	0.4109	4.7702	0.2096
Diallyl sulfide (DAS)	-	-	1.3896	0.7196	-	-
Methyl allyl disulfide (AMS)	-	-	1.1860	0.8431	-	-
*S*-Allyl-l-cysteine (SAC) (*S*-1-Propenyl-l-cysteine)	1.5925	0.6280	128.2727	0.0078	-	-
*S*-Methyl-l-cysteine (SMC)	1.7681	0.5656	127.0000	0.0079	-	-
*S*-Methyl-l-cysteine sulfoxide (SMCS)	0.5857	1.7074	0.7755	1.2896	0.7361	1.3585
*S*-Methyl mercapto-l-cysteine (SMMC)	0.2545	3.9290	1.2378	0.8079	0.3569	2.8016
Total	0.6308	1.5852	1.0545	0.9483	1.6271	0.6146
	**SS**		**ES**		**IS**	
**Saponins**	**White/Purple**	**Purple/White**	**White/Purple**	**Purple/White**	**White/Purple**	**Purple/White**
Agapanthagenin	-	-	0.0031	320.2470	-	-
β-Chlorogenin	0.6367	1.5705	0.0130	76.9899	1.0887	0.9185
Desgalactotigonin	0.1966	5.0872	0.0101	98.5283	-	-
Desgalactotigonin-rhamnose	-	-	-	-	-	-
Diosgenin	0.7670	1.3037	0.0105	95.3023	-	-
Eruboside B	1.3198	0.7577	0.0156	64.0878	-	-
Eruboside B-rhamnose	0.1158	8.6331	0.0267	37.3852	-	-
Gitogenin	0.6642	1.5056	0.0130	77.0948	1.0887	0.9185
Proto-desgalactotigonin	-	-	-	-	-	-
Proto-desgalactotigonin-rhamnose	-	-	-	-	-	-
Proto-eruboside B	0.1705	5.8666	0.0034	293.7059	2.0534	0.4870
Sativoside B1-rhamnose	-	-	-	-	-	-
Sativoside R1	-	-	-	-	-	-
Sativoside R2	0.2160	4.6288	0.0157	63.5507	0.9527	1.0497
Sativoside R2-rhamnose	0.0145	69.0498	0.0116	86.4215	-	-
Voghieroside D1	0.2191	4.5643	-	-	-	-
Voghieroside E1	-	-	0.0147	67.9012	5.1114	0.1956
Total	0.5540	1.8051	0.0135	73.8849	1.4165	0.7059

## Supplementary Materials

Supplementary Materials are available online.
