# Supplementary material for: Tissue-Specific Accumulation of Sulfur Compounds and Saponins in Different Parts of Garlic Cloves from Purple and White Ecotypes
Source: Molecules. 2017 Aug 20;22(8):1359. doi: 10.3390/molecules22081359 (PMC6152257; doi:10.3390/molecules22081359)
Supplement: Supplementary file 1 [file molecules-22-01359-s001.pdf]

Supplementary Materials

Tissue-Specific Accumulation of Sulfur Compounds and Saponins in Different Parts of Garlic Cloves from Purple and White Ecotypes

Gianfranco Diretto [1], Angela Rubio-Moraga [2], Javier Argandoña [2], Purificación Castillo [3], Lourdes Gómez-Gómez [2] and Oussama Ahrazem [2,4,*]

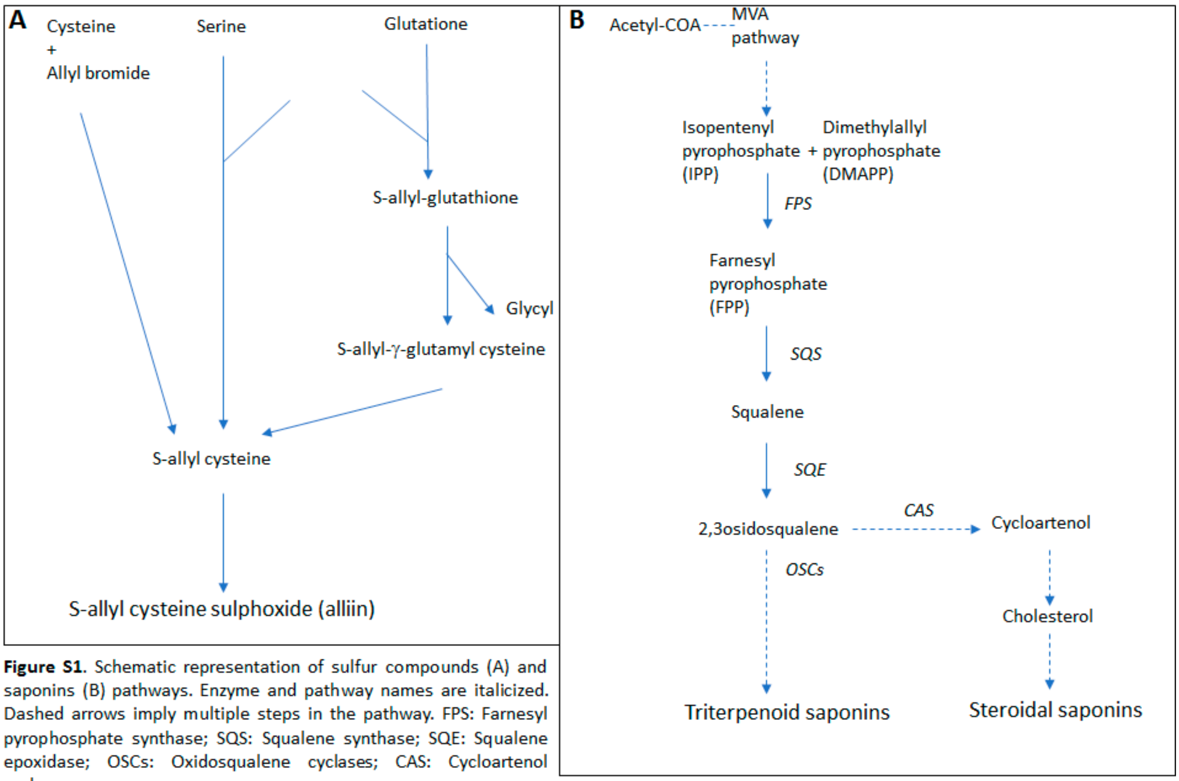

Figure S1. Schematic representation of sulfur compounds (A) and saponins (B) pathways. Enzyme and pathway names are italicized. Dashed arrows imply multiple steps in the pathway. FPS: Farnesyl pyrophosphate synthase; SQS: Squalene synthase; SQE: Squalene epoxidase; OSCs: Oxidosqualene cyclases; CAS: Cycloartenol cyclase.

Table S1. LC-HRMS analyses of sulfur compounds in three tissues (SS, ES and IS) of white and purple garlic ecotypes. Data are expressed as AVG+ST.DEV. AVGs, which represent, for each metabolite, the fold over the internal standard (IS) have been obtained by using, at least, 3 independent biological replicates. Asterisks indicate significance of the fold change in an ANOVA plus Tukey's t-test (*: $p \leq 0.05$;**: $p \leq 0.01$; ***: $p \leq 0.001$).

	WHITE-SS	PURPLE-SS	WHITE-ES	PURPLE-ES	WHITE-IS	PURPLE-IS
**2-vinyl-2,4-dihydro-1,3-dithiin**	0.0028±0.0001	1.8123±0.0003***	0.0082±0.0015	0.0118±0.0007	0.0005±0.0001	0.0008±0.0001*
**ajoene**	0.0756±0.0020	2.7861±0.0048***	0.1318±0.0065	0.1815±0.0103*	0.0075±0.0004	0.0088±0.0010
**allicin**	0.5215±0.0001	3.3462±0.0006***	2.2306±0.1522	3.7611±0.2797**	0.4038±0.0054	nd
**alliin**	5.9797±0.0943***	0.2654±0.0597	7.5575±0.2785**	5.8623±0.0399	0.8561±0.0105	0.7598±0.0469
**allitridin**	0.0002±0.0001	nd	0.4448±0.0002***	0.0039±0.0005	0.0001±0.0001	0.0001±0.0001
**diallyl disulfide**	0.0029±0.0004	2.2207±0.2014***	0.0036±0.0001*	0.0015±0.0002	nd	nd
**diallyl sulfide**	nd	nd	0.0007±0.0001	0.0005±0.0001	nd	nd
**methyl allyl disulfide**	nd	nd	0.0002±0.0001	0.0001±0.0001	0.0001±0.0001	nd
**s-allyl-L-cysteine (SAC) (S-1-Propenyl-l-cysteine)**	0.5209±0.0295*	0.3271±0.0171	0.2822±0.0153***	0.0022±0.0004	nd	nd
**s-methyl-L-cysteine (SMC)**	0.0122±0.0013*	0.0069±0.0003	0.0508±0.0027***	0.0004±0.0001	nd	nd
**s-methyl mercapto-L-cysteine**	0.0001±0.0001	0.0006±0.0001**	0.0006±0.0001	0.0005±0.0001	0.0003±0.0001	0.0008±0.0001**
**s-methyl-L-cysteine sulfoxide**	0.0341±0.0036	0.0582±0.0054*	0.0527±0.0094	0.0679±0.0033	0.0139±0.0016	0.0188±0.0009*
**TOTAL**	7.1500±0.1211	10.8235±0.0768**	10.7637±0.4622	9.8937±0.4106	1.2823±0.0149**	0.7891±0.08102

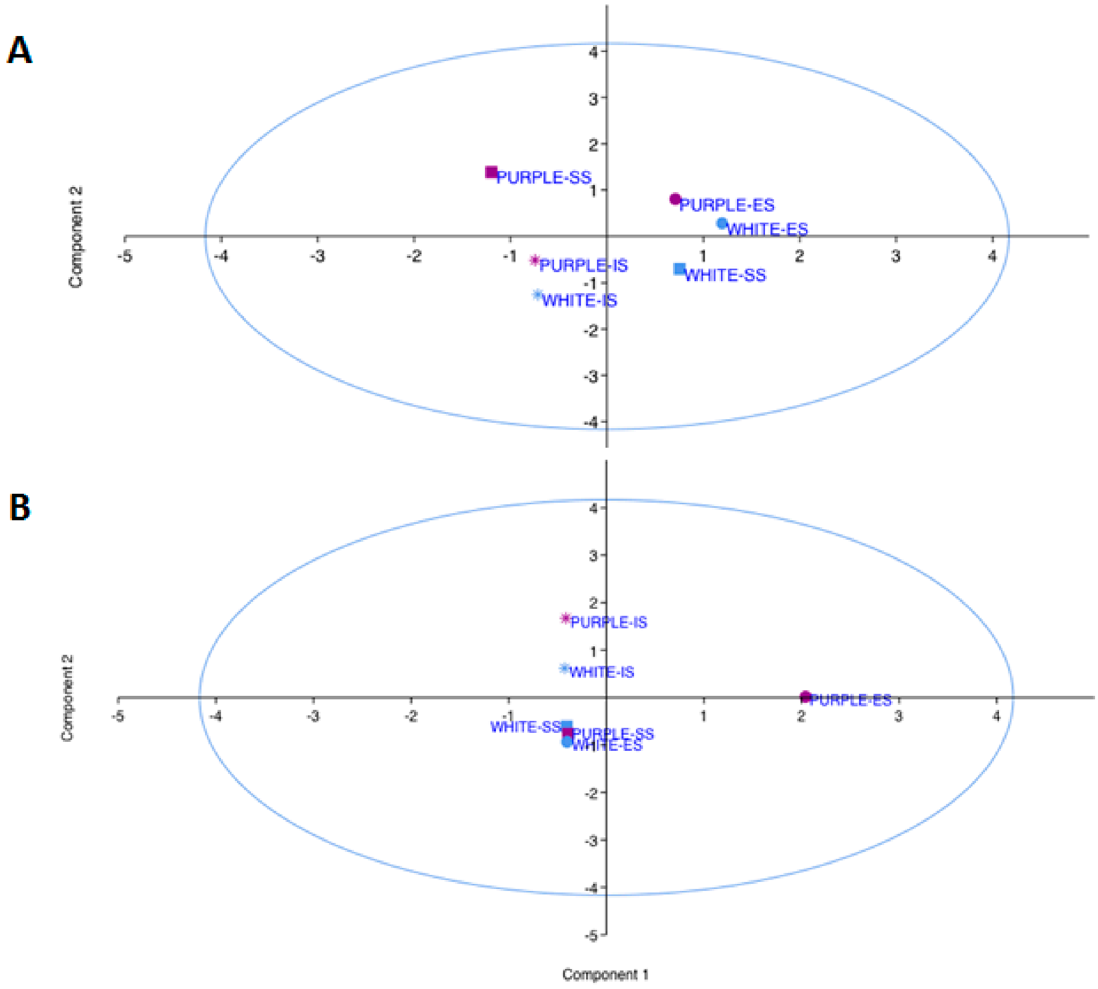

Figure S2. Principal component analysis (PCA), according the tissues/ecotypes, of sulfur compounds (A) and saponins (B) detected in three tissues (SS, ES and IS) of the white and purple ecotypes of garlic. Data represent, for each metabolite, the fold over the internal standard (IS) intensity.

Table S2. LC-HRMS analyses of saponin compounds in three tissues (SS, ES and IS) of white and purple garlic ecotypes. Data are expressed as AVG+ST.DEV. AVGs, which represent, for each metabolite, the fold over the internal standard (IS) have been obtained by using, at least, 3 independent biological replicates. Asterisks indicate significance of the fold change in an ANOVA plus Tukey's t-test (*: $p \leq 0.05$;**: $p \leq 0.01$; ***: $p \leq 0.001$).

	WHITE-SS	PURPLE-SS	WHITE-ES	PURPLE-ES	WHITE-IS	PURPLE-IS
**agapanthagenin**	nd	0.0010±0.0003	0.0009±0.0002	0.2928±0.0209***	nd	nd
**b-chlorogenin**	0.0536±0.0123	0.0841±0.0092*	0.0654±0.0153	5.0366±0.5253***	0.0011±0.0003	0.0010±0.0002
**desgalactotigonin**	0.0005±0.0001	0.0025±0.0006**	0.0011±0.0002	0.1043±0.0112***	nd	0.0009±0.0002
**desgalactotigonin-rhamnose**	nd	nd	nd	0.0018±0.0001	nd	nd
**diosgenin**	0.0086±0.0020	0.0111±0.0009	0.0088±0.0019	0.8386±0.0506***	nd	nd
**eruboside B**	0.0461±0.0062	0.0349±0.0058	0.0140±0.0040	0.8999±0.1672***	0.0002±0.0001	nd
**eruboside B-rhamnose**	0.0076±0.0010	0.0654±0.0071***	0.0171±0.0026	0.6389±0.1680***	0.0002±0.0001	nd
**gitogenin**	0.0536±0.0123	0.0806±0.0088	0.0652±0.0153	5.0270±0.5245***	0.0011±0.0003	0.0010±0.0002
**proto-desgalactotigonin**	nd	nd	nd	0.0004±0.0001	nd	nd
**proto-desgalactotigonin-rhamnose**	nd	0.0003±0.0001	nd	nd	nd	nd
**proto-eruboside B**	0.0004±0.0001	0.0024±0.0006***	0.0002±0.0001	0.0446±0.0107***	0.001±0.0001	0.0005±0.0002*
**sativoside B1-rhamnose**	nd	nd	nd	0.0026±0.0002	nd	nd
**sativoside R1**	nd	nd	nd	0.0014±0.0004	nd	nd
**sativoside R2**	0.0064±0.0002	0.0296±0.0031***	0.0134±0.0020	0.8516±0.2098***	0.0001±0.0001	0.0001±0.0001
**sativoside R2-rhamnose**	0.0001±0.0001	0.0063±0.0003***	0.0003±0.0001	0.0274±0.0076***	nd	nd
**voghieroside D1**	0.0001±0.0001	0.0003±0.0001**	nd	0.0046±0.0007	nd	nd
**voghieroside E1**	nd	0.0005±0.0001	0.0001±0.0001	0.0074±0.0021***	0.0016±0.0001***	0.0003±0.0001
**TOTAL**	0.1768±0.0342	0.3192±0.0368***	0.1865±0.0416	13.7800±1.6995***	0.0054±0.0007	0.0038±0.0008

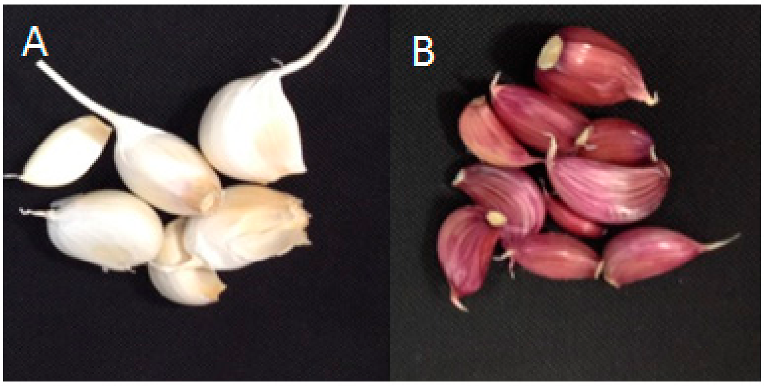

Figure S3. White (A) and purple (B) garlic ecotypes under study.
